# COVID-19 Vaccination: Sociopolitical and Economic Impact in the United States

**DOI:** 10.3390/epidemiologia3040038

**Published:** 2022-11-08

**Authors:** Soyoung Jeon, Yu-Feng Lee, Komla Koumi

**Affiliations:** Department of Economics, Applied Statistics and International Business, New Mexico State University, Las Cruces, NM 88003, USA

**Keywords:** COVID-19 vaccination, socioeconomic–political status, racial and ethnic disparities, public health policy, GIS

## Abstract

Since the outbreak of COVID-19, vaccination against the virus has been implemented and has progressed among various groups across all ethnicities, genders, and almost all ages in the United States. This study examines the impacts of socioeconomic status and political preference on COVID-19 vaccination in over 443 counties in the southwestern United States. Regression analysis was used to examine the association between a county’s vaccination rate and one’s personal income, employment status, education, race and ethnicity, age, occupation, residential area, and political preference. The results were as follows: First, counties with higher average personal income tend to have a higher vaccination rate (*p* < 0.001). Second, county-level vaccination is significantly associated with the percentage of Democrat votes (β = 0.242, *p* < 0.001). Third, race and ethnicity are vaccine-influencing factors. Counties with more Black residents have lower vaccine acceptance (β = −0.419, *p* < 0.001), while those where more Hispanics or Native Americans reside are more likely to accept vaccines for health protection (β = 0.202, *p* < 0.001; β = 0.057, *p* = 0.008, respectively). Lastly, pertaining to the age difference, seniors aged 65 and older show substantial support for vaccination, followed by the median age group (all *p* < 0.001).

## 1. Introduction

On 11 March 2020, when the World Health Organization (WHO) declared COVID-19 a global pandemic, the so-called ‘World War III on Coronavirus’ prompted countries worldwide to announce their respective national emergencies to fight against the ‘global enemy’ by mandating health-ensuring protocols (e.g., face-covering and social distancing), international travel closures, business and institutional lockdowns, or limited operation, and most importantly researching and developing effective COVID-19 vaccines for crisis control. Facing COVID’s ‘hard landing,’ as early as early December 2020 and January 2021, the ‘COVID-19 vaccine race’ kicked off the first vaccination in the UK and Israel, followed by the US and many others in the common goal of demolishing the ‘enemy’ [1].

Following individual or joint efforts and extended attempts, as many as 21 COVID-19 vaccines, led by Moderna and Janssen/Johnson & Johnson of the US, AstraZeneca of the UK, Pfizer/BioNTech of Germany, and Sinovac and CanSino Biologics Inc. of China, have been launched in countries globally covering 60.3% (or 5 billion) of the world population with at least one dose injection since January 2022. Momentously, this adds up to a total of 9.8 billion doses already administered, while currently 28.8 million daily shots are reported to be given to increase the vaccine coverage [1,2]. Since the global roll-out, countries receiving high vaccination coverage vary greatly in their levels of economic development and social and political arrangements, including the United Arab Emirates at 92% full vaccination and 7% part vaccination (or, total at 99%), Portugal at 90% and 4%, respectively (or, total at 94%), Cuba at 93% (total), Chile at 91% (total), Singapore 89% (total), China at 88% (total), Canada at 84% (total), followed by Italy, Japan, France in between before Vietnam at 79% (total), Brazil at 78% (total), the UK at 76% (total), and the US at 75% (total) alongside Germany heading the rest of the world. Nonetheless, extremely low vaccination rates (at least one dose administered) of 9.4% have been observed across lower-income and underdeveloped nations, where Ethiopia and Nigeria (7.9% and 6.2% of total coverage, respectively) were victims of the frailty in their economies and politics. As recently emphasized by the WHO in promoting the goal of ‘vaccine equity’, “No one is safe from COVID-19 until everyone is safe” since the disease is believed to be under control or possibly ended only when vaccination is non-discriminatory across all countries from the most powerful to the most vulnerable. Given WHO’s target of 70% global vaccination presumed necessary for herd immunity, and facing the pressure of a virus moving faster than the global vaccine distribution, it is a global priority task to reallocate, fairly distribute, and administer the vaccines from countries with a surplus to those experiencing a shortage to prevent the pandemic from worsening [3].

Within the United States, President Biden’s administration has pledged a national effort in the struggle against COVID-19. Since early 2021, the COVID-19 vaccination race has encompassed various groups across all ethnicities, genders, and almost all ages. As of January 2022, including the initial and booster shots, high (full) vaccination has been reached in the US Northeast (e.g., the states of New York (74%), Pennsylvania (66%), Virginia (70%), and New England (average over 72%)), the west coast (e.g., the states of Washington (70%), Oregon (68%), California (68%)), and a couple of Mid- and Southwest states, namely New Mexico (68%) and Colorado (68%). Their lower-rate (at or below 55%) counterparts are typically spread across the central-eastern coastal regions (e.g., South Carolina (55%), Georgia (53%)), the southern Midwest (e.g., Alabama (49%), Mississippi (50%), Oklahoma (55%)), and Mid- and Northwest (e.g., Wyoming (50%), Idaho (53%), North Dakota (54%)), leaving the ‘medium achievers’ such as Arizona (59%), Texas (59%), and Nevada (59%) [4]. Given such asymmetric cross-state coverage, this paper investigates whether and how the COVID-19 vaccination is affected by macroeconomic and sociopolitical factors such as personal income, employment status, education, race and ethnicity, age, occupation, rural vs. urban dwelling, and political preference across 443 counties in the states of Arizona, Colorado, New Mexico, Oklahoma, and Texas.

Since the outbreak of COVID-19, studies across almost all fields and dealing with almost all aspects of the public health crisis have appeared in record numbers in the literature. From the debates over the origin of the virus to the effects of COVID-19 vaccination more recently, the intellectual development and exchange have been dynamic, innovative, and prolific. Given this analysis centering on the context of COVID-19 vaccination, relevant literature is summarized in the subsequent subsections.

### 1.1. Mentality on COVID-19 and Attitude toward Vaccine Acceptance

Internationally, the public acceptability and attitude toward the COVID-19 vaccine have been reported as being low. El-Elimat et al. [5] attested to the case of Jordan, where low acceptance is rooted in concerns over vaccine safety following incomplete, non-transparent, or misleading vaccine information (also see [6,7,8]). Likewise, Machingaidze and Wiysonge [9] claimed ‘vaccine hesitancy’ in the study of low- and middle-income countries across Africa, South Asia, and Latin America. Although vaccine acceptability may seem to vary in those countries, higher acceptance is believed only to occur when vaccine safety is high, with low or no vaccination cost, and the vaccine-related information is trustworthy. As emphasized in the Oxford Coronavirus Explanations, Attitudes, and Narratives Survey (OCEANS-III), personal over the collective benefit of vaccination should be a key driving factor in increasing group immunity [10]. In Chile, COVID-19 vaccine refusal or indecision (as ‘vaccine hesitancy’) has also been noted, reflecting concerns about the vaccine’s side effects and effectiveness, the perceived benefits for the injected and his community, the availability of disease and vaccine information, and the pandemic trend [11,12,13]. Similarly, Majeed et al. [14] alerted that the lack of data and analysis on long-term vaccine safety and efficacy tends to contribute to British ‘vaccine hesitancy’. As claimed by Cascini et al. [15], global herd immunity against COVID-19 could be impeded by vaccine hesitancy across countries, including barriers of negative vaccine perception (e.g., efficacy, safety, and price), especially among those with lower income and educational level, not medically insured, and rural-dwelling, as well as self-identified ethnic minorities. Kaplan and Milstein [16] suggested that in the US, “vaccine acceptance improved when the efficacy increased beyond 70%”, contrary to a low acceptance when the probability of severe adverse reactions ascended.

### 1.2. COVID-19 Vaccine Safety and Efficacy 

Facing acute and highly transmissible COVID-19, vaccine safety and efficacy are crucial factors a country’s citizens assess before their vaccination decisions. Polack et al. [17] reported that a two-dose COVID-19 vaccine by BNT162b2 mRNA reached 95% protection in persons 16 years and older while severe side effects (e.g., shortness of breath or chest pain) were low and comparable to other vaccines and placebo groups (also see [18]). In the same perspective, some studies have produced equally comprehensive reports, including different vaccines under respective production platforms (e.g., live attenuated vaccine (LAV); inactivated virus vaccine; sub-unit vaccine; DNA vaccines, and RNA vaccines) with specific advantages and limitations [19,20]. Collectively, it offers vaccine developers a key reference, and general vaccine information on short-term vaccine efficacy and safety to the public. Alternatively, Rodrigues and Plotkin [21] identified health, economic, and social benefits upon the receipt of COVID-19 vaccination, suggesting broader immunization to alleviate the pandemic.

### 1.3. Socioeconomic and Sociopolitical Impact on COVID-19 Vaccination 

Ironically, the coronavirus brings about not just a purely medical catastrophe. Its complexity is rather all-encompassing, involving socioeconomic conditions and politics, which impact the outcome of vaccination. Boserup et al. [22], for instance, uncovered the disproportionate impact of COVID-19, with higher virus infections and deaths, on racial and ethnic minorities in the US, alerting the need for vaccination across these minorities to stem the spread. García and Cerda also suggested the socioeconomic characteristics of the community be considered so as to implement an adequate and proper public health policy for COVID-19 control [12]. Recent studies have disclosed that the ‘political divide’ along with income and racial disparities affected COVID-19 casualty and vaccination rates, as Republican voters were less willing to be vaccinated, leading to significantly higher disease cases and deaths [23,24,25]. Similar evidence of COVID-19 being a politicized issue is also revealed in social and public media such as Twitter [26]. While Blacks tended to express reservations about vaccine safety and efficacy during the 2009 H1N1 influenza pandemic [27], structural racism and ethnic inequities against minorities such as Hispanics and Black Americans hampered the overall COVID-19 vaccination rate, pointing to possible social structural change as a means to improve immunization across all races [28,29].

This study aims to outline the socioeconomic and sociopolitical behavior of regional citizens reacting to the COVID-19 vaccination in hopes of offering local, state, and national policymakers a better understanding to properly prepare and strategize the next steps or plans to end or coexist with the disease. The paper is structured as follows: The Section 1 starts with an ‘Introduction’. The Section 2 summarizes the ‘Materials and Methods’. The Section 3 of ‘Results’ analyzes the empirical findings. The Section 4 offers ‘Discussion and Policy Implications’, followed by the Section 5 ‘Conclusions’.

## 2. Materials and Methods

### 2.1. Data Sources and Variables

County-level COVID-19 vaccination rates, counting adults aged ≥18 years who were fully vaccinated (either with the Pfizer-BioNTech or Moderna vaccine, or a single dose of the Johnson & Johnson vaccine) as of 1st May 2021, were analyzed in this study. The analyses are based mainly on five sources of data: Bureau of Labor Statistics, Bureau of Economic Analysis, 2010 US Census, Politico, and Centers for Disease Control and Prevention (CDC), covering an aggregated dataset of a total of 443 counties across all five states (AZ, CO, NM, OK, and TX) in the Mid- and Southwest from the outbreak of COVID-19 until mid-2021. Various independent variables covering socioeconomic and political aspects at the county level with respective data sources were included, as described below and shown in Table 1.

- *State*: Five states (Arizona, Colorado, New Mexico, Oklahoma, and Texas) across the United States.
- *Employment status*: The unemployment rate of 2019 is used to denote county-wide employment status, retrieved from the Bureau of Economic Analysis. The expectation is that it will disclose an inverse correlation between a county’s unemployment rate and its COVID-19 vaccination rate. That is, citizens who are unemployed may be inclined to refuse vaccination due to economic reasons or not being subject to the workplace vaccination requirement.
- *Political choice*: The debates over the political ideology of COVID-19 suggested potential polarization over the vaccination decision. Hence, county-based voting results of the 2020 presidential election released by Politico (https://www.politico.com/2020-election/results/, accessed on 15 June 2021) were analyzed. In brief, it is hypothesized that counties with a high proportion of Democratic voters would be more likely to get vaccinated.
- *Age*: As COVID-19 vaccination was authorized on an age basis, various age classifications were used in this study following the county data from ‘County Population by Characteristics: 2010–2019’ of the 2010 US Census. As guided by CDC’s Advisory Committee on Immunization Practices (ACIP) [30], COVID-19 vaccines were first administered to healthcare personnel and long-term care facilities’ residents categorized as Phase 1a, and almost concurrently given to the seniors aged ≥75 as Phase 1b, followed by Phase 1c covering persons aged 65–74 years and those aged 16–64 years with high-risk medical conditions. Based on such categorization, the age groups in this analysis were structured as shown in Table 1.
- *Occupation*: Worker’s occupation is classified into ‘farm’ and ‘non-farm’ categories from the 2019 data of the Bureau of Economic Analysis, where the percentage of farm employment (labeled as ‘Farm Worker’ in the model) is calculated by taking the number of workers hired as farm labor over the total population per county. The assumption is that counties with more farmworkers are more likely to have lower COVID-19 vaccination rates.
- *Area of residence*: We examine whether the COVID-19 vaccination rate varies in the area of residence by extending the model to cover the rural and urban communities based on the 2010 Census data. Counties with less than 50% of the population living in rural areas are classified as urban, in contrast to those with 50-plus percent of rural residents. As indicated in the recent Morbidity and Mortality Weekly Report (MMWR) of the CDC [31], access to COVID-19 vaccines is normally lower, at 38.9%, in rural counties than in cities. This study, therefore, assumes a lower vaccination rate across rural areas.
- *Education*: The education level also serves as a determinant for COVID-19 vaccination. Soares et al. [32] found that individuals with secondary or basic (lower) education and the uneducated are more hesitant to get vaccinated, resulting in lower vaccination rates compared to those with college or university degrees. Based on Federal Reserve Economic Data, the percentage of the county population aged ≥18 who had obtained at least a high-school diploma was examined for the education effect on the vaccination choice. It is projected that a lack of college education would lead to county citizens’ vaccination hesitancy.
- *Income*: The COVID-19 pandemic has created much economic hardship for most people, as different income levels may potentially increase the acceptance or refusal of the vaccine. This study hence tries to disclose the effect of per capita income on vaccination following the data from the Bureau of Economic Analysis, with the fundamental prediction resting on the higher lags in vaccination rates among counties with lower income.
- *Race and ethnicity*: Race and ethnicity are exogenous variables and important indicators in this analysis which aims to offer policy implications, possibly reducing racial and ethnic disparities to improve overall COVID-19 vaccine coverage across five states. CDC identified racial and ethnic discrimination, amid other factors, creating challenges to COVID-19 vaccination access and acceptance among minority groups [33]. Thus, it is assumed that lower vaccination rates prevail in counties dominated by minorities of Blacks, Asians, Native Americans, and Hispanics, diverging from those predominantly inhabited by Whites and non-Hispanics.

### 2.2. Statistical Method

**Figure 1:**
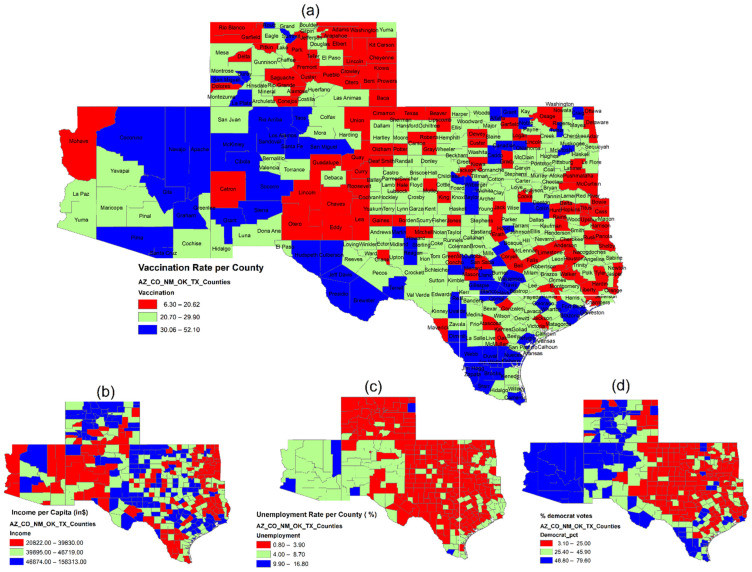
Geographic distribution of (**a**) COVID-19 vaccination rate as of 1 May 2021 and economic status, which is described by (**b**) income per capita; (**c**) unemployment rate; (**d**) political affiliation.

**Figure 2:**
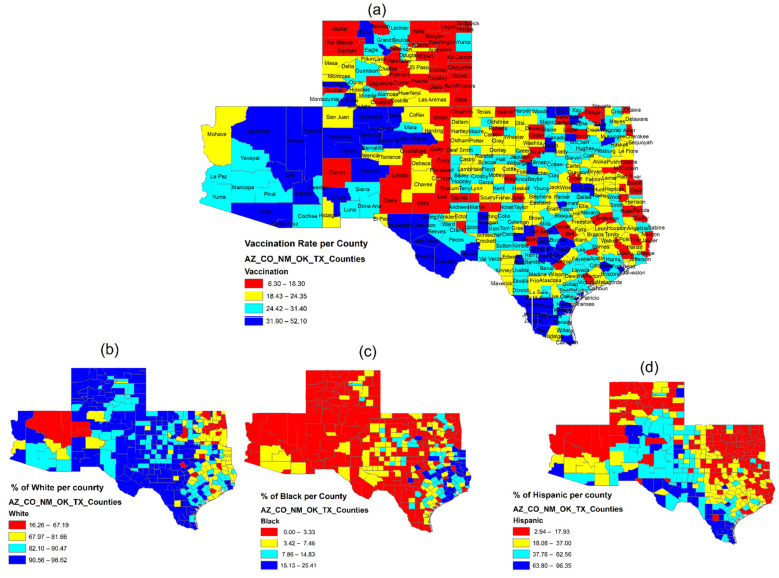
Geographic distribution of (**a**) COVID-19 vaccination rates as of 1 May 2021, and race and ethnicity effects; (**b**) percent of the White population, (**c**) percent of the Black population, and (**d**) percent of the Hispanic population.

A preliminary summary of county-level factors and vaccination rates was analyzed in Table 2. ArcGIS (version 10.8) was used to map the spatial distribution of the vaccination rates and show how socioeconomic and political variables impacted COVID-19 vaccination across counties and states (see Figure 1 and Figure 2).

**Figure 3:**
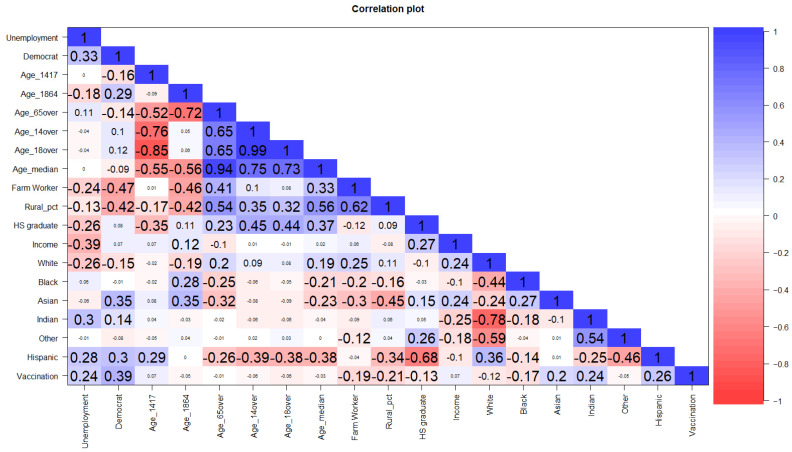
Pairwise correlations among socioeconomic variables. All significant correlations are expressed in bold.

Linear regression analysis was used to examine associations of socioeconomic factors with county-level vaccination rates. Based on the multicollinearity detection using variance inflation factors (VIF) and correlation plot (see Figure 3), seven variables were selected for inclusion in the default model, as follows:

(1) $$M1:\;\mathrm{vaccination}\mathrm{rate}=\beta_{0}\;+\;\beta_{1}\mathrm{state}+\beta_{2}\mathrm{unemployment}+\beta_{3}\mathrm{democrat}+\;\beta_{4}\mathrm{farmworker}+\beta_{5}\mathrm{rural}\_\mathrm{pct}+\beta_{6}\mathrm{HS}\mathrm{graduate}+\beta_{7}\mathrm{income},$$

where $\beta_{0}$ refers to the intercept of the vaccination rate and $\beta_{i}$ denotes the coefficients associated with the covariates (*i* = 1, 2, ⋯, 7). We then added each age variable (Age_1417, Age_1864, Age_65over, Age_14over, Age_18over, and Age_median) to Model 1 to examine the adjusted effects by population percent of various age groups. Model 2 has the following formula:

(2) $$M2:\;\mathrm{vaccination}\mathrm{rate}=\beta_{0}\;+\;\beta_{1}\mathrm{state}+\beta_{2}\mathrm{unemployment}+\beta_{3}\mathrm{democrat}+\;\beta_{4}\mathrm{farmworker}+\beta_{5}\mathrm{rural}\_\mathrm{pct}+\beta_{6}\mathrm{HS}\mathrm{graduate}+\beta_{7}\mathrm{income}+\beta_{8}\mathrm{age},$$

where $\beta_{8}$ refers to the coefficient of each age-specific variable. Table 3, Tables S1 and S2 show the estimated coefficients obtained for the model with Age_65over, Age_1864, and Age_median, respectively. As the next stage, each race-specific variable (White, Black, Asian, Indian, and Hispanic population percent) was added to Model 2 to explore the race/ethnicity-specific relationship with the vaccination rate, as given in Model 3:

(3) $$M3:\;\mathrm{vaccination}\mathrm{rate}=\beta_{0}\;+\;\beta_{1}\mathrm{state}+\beta_{2}\mathrm{unemployment}+\beta_{3}\mathrm{democrat}\;+\;\beta_{4}\mathrm{farmworker}+\beta_{5}\mathrm{rural}\_\mathrm{pct}+\beta_{6}\mathrm{HS}\mathrm{graduate}+\beta_{7}\mathrm{income}+\beta_{8}\mathrm{age}+\;\beta_{9}\text{race/ethnicity},$$

where $\beta_{9}$ refers to the coefficient associated with each race/ethnicity variable. The separate model for each racial and ethnic variable was identified (M3.1)–(M3.5) in Table 3, Tables S1 and S2. All statistical analyses were conducted using the software R (version 4.1.0). The level of significance was set at a *p*-value of <0.05.

## 3. Results

Figure 1 illustrates relationships between socioeconomic and political affiliations and COVID-19 vaccination rates across the Mid/Southwest region. Overall vaccination rates as of 1st May 2021 ranked from highest to lowest by state were Arizona (33%), New Mexico (26%), Texas (25%) and Oklahoma (25%), and Colorado (20%). In sum, the likelihood of vaccine acceptance was found to be positively associated with county-level personal income and the level of unemployment. It suggests that county citizens with higher personal income would tend to seek vaccine protection under the rationale that vaccination is widely believed to help one keep his/her job which then secures his/her income. Likewise, the unemployed across counties and states would tend to be vaccinated in order to use the proof of vaccination to find their next employment. Alternatively, the map of political preferences shows a pattern of higher vaccination rates in counties with a higher percentage of Democrat votes than of Republicans, which is especially true for the ‘Democrat’ counties including Apache (vaccination rate: 46.9%) and Santa Cruz (45.2%) in Arizona and McKinley (50.6%), Cibola (38.8%), and Los Alamos (38.4%) in New Mexico. As a mirror reflection, the highest correlation of vaccination rate and Democratic affiliation was observed at *r* = 0.39, as shown in Figure 3. This finding aligned with the claim of Bardosh et al. [34] which emphasized that political and social polarization may end up with reverse causation leading to adverse vaccination effects.

Figure 2 indicates that county vaccination rates correlate with percentages of the county population by race and ethnicity. In general, there appears to be a negative relationship between vaccination rate and the Black population, while there is a positive correlation with Hispanic groups. This result resonates with the study of Kricorian and Turner [35] in a recent vaccination survey, claiming that Black Americans “were less likely to want the COVID-19 vaccine at all compared with Whites and Hispanics” and “mistrust of the vaccine among Black respondents was significantly higher than other racial/ethnic groups”. Meanwhile, the latest data on COVID-19 vaccination by race/ethnicity corresponding to CDC reports indicates that by 31 January 2022, 74% of Americans across all races and ethnicities had received at least one dose of a COVID-19 vaccine [36]. Among them, Whites, who represent 61% of the total population, make up 56% of people with at least one dose. Hispanic citizens, who represent 17% of the total population, reach 20% of the one-dose vaccinated, while Blacks, who represent 12% of the total population, make up just to 10% of individuals who received at least one-dose, followed by other minorities, such as Asians at 6% of the total population and 7% of the single-shot group. In the correlation plot of Figure 3, the vaccination rate was lower among counties with higher proportions of Whites (*r* = −0.12) and Blacks (*r* = −0.17), while opposite relationships were observed for Asians (*r* = 0.2), Native Americans (*r* = 0.24), and Hispanics (*r* = 0.26). This indicates that across the five states, Hispanic residents may be more inclined than Blacks and other minorities to accept virus protection and choose vaccination. Weak correlations were found between race/ethnicity and socioeconomic factors, except for the negative association between the percentage of high school graduates and Hispanics (*r* = −0.68).

Table 2 includes a preliminary summary containing the descriptive statistics for the socioeconomic and political variables and vaccination rates across all 443 counties and the respective number of counties within each state. As of May 2021, the mean of county-level vaccination rate in the Mid- and Southwest regions was 24.59%, with the lowest (19.63%) in Colorado and the highest (32.81%) in Arizona. In particular, counties with higher average vaccination rate in Arizona had a higher percentage of Democrat votes (62.5%) and a population aged ≥65 years (21.7%), while these counties were endowed with the lowest population of farm workers (1.58%), the lowest percentage of rural population (34.11%), and the lowest income per capita ($39,197). On the contrary, Colorado’s counties, on average attaining the lowest state-wide COVID-19 vaccination rates, showed the highest income per capita ($53,080), the highest percentage of Whites (91.83%), the most high-school graduates (91.16%), but the lowest unemployment rate (2.74%). With regard to such socioeconomic vaccination differences between Arizona and Colorado, it is somewhat evident that with the comparable number of Black residents in both states (i.e., 2.36% in Arizona and 2.01% in Colorado), the vaccine coverage among Whites in Colorado seemed deficient and lingering as compared with Arizona. Moreover, Colorado had significantly more farm workers (5.52%) and a greater proportion of rural land (58.68%) than Arizona (1.58% and 34.11%, respectively). This could logically be linked to its lower state-wide vaccination rate since its farm labor may disperse across more extensive and remote rural areas facing less worrisome virus transmission. Lastly, in the 2020 presidential election, states with more counties that voted for the Democrats, for example, Arizona (100%, obtained from *Democrat_1*) and New Mexico (42.4%), tended to have higher vaccination rates. States with more counties classified as urban areas, such as Arizona (80%, calculated from *Urban_1*) and New Mexico (63.6%), were more likely to have higher vaccination rates. Hypothetically, the higher urban vaccination rate reflects the population cluster effect as city people would receive vaccines to avoid virus infection due to their relatively close contact with others.

Key regression results from different model specifications between the county-level characteristics and vaccination rates are described in Table 3. In model (1), it is observed that the county’s state, Democratic affiliation, and income per capita were significant factors influencing the percentage of those vaccinated in the county (*R*^2^ = 0.382). The ‘State’ variable was shown to be an important categorical factor in predicting vaccination rates for respective counties because each county has the characteristic of being dependent on the state as its administrative subdivision. Increases in vaccination rates were associated with a higher percentage of the Democratic vote and per capita income ($\beta_{3}$ = 0.242, $\beta_{7}$ = 8.077e-05; all *p* < 0.001).

**Figure 4:**
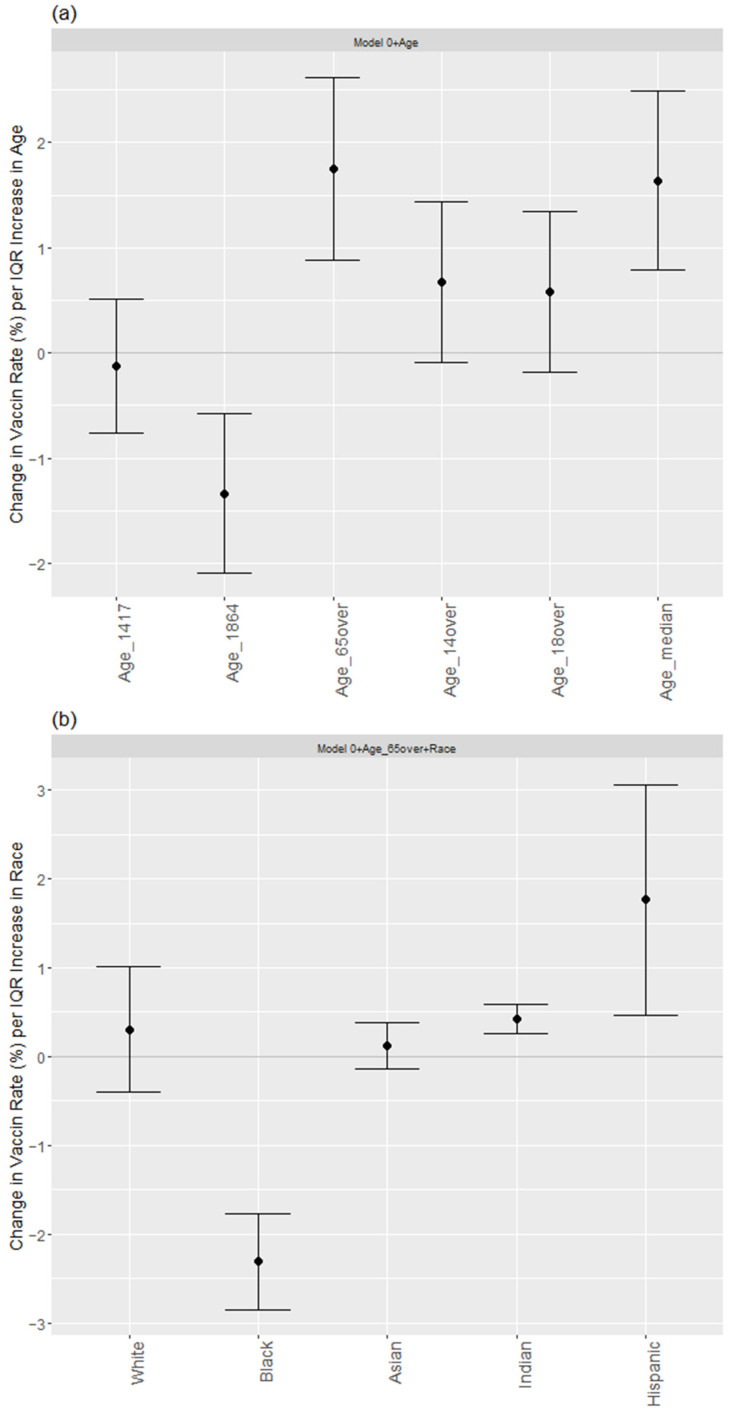
(**a**) Change in vaccination rate (%) per interquartile range (IQR) increase in each age variable; (**b**) Change in vaccination rate (%) per interquartile range (IQR) increase in each race/ethnicity variable.

When each age variable was added to the model (1), the increase in the population aged 65 years or older had the most substantial and positive effect on the change in the vaccination rate among other age variables, followed by a significantly negative impact on the change in the vaccination rate in the age group of 18–64 years, and a significantly positive effect on the change in the vaccination rate with respect to the median age group, as the corresponding estimates and 95% confidence intervals are shown in Figure 4a. That is, model (2) containing individuals aged ≥65 years performed the best by explaining 40.4% of the total variation in vaccination rates (*R*^2^ = 0.404, see Table 3). In general, the vaccination rate would *increase* by 0.245% per one percent increase in the elderly population in a county (*p* < 0.001). Nonetheless, as the model included a population aged between 18–64 years old, it showed that the vaccination rate would *decrease* by 0.265% per one percent increase in this age group (*p* = 0.001, *R*^2^ = 0.399 in Table S1). Finally, when the model was adjusted for the median age, it was found that the vaccination rate would increase by 0.222% for a one-year increase in the median age of the county population (*p* < 0.001, *R*^2^ = 0.402 in Table S2).

Given the above age–vaccination factor, the model corrected for the age ≥ 65, which produced the most significant effect on the COVID-19 vaccination rate, was selected. Each race variable was added to M2 (2) to explore the effect of specific county-based racial/ethnic densities on vaccination, as tabulated in models M3.1–M3.5 of Table 3. The calculated VIF indicated no significant multicollinearities between the race/ethnic variable and socioeconomic factors, particularly for the correlation between Hispanic and high school graduate percentages in M3.5. Explicitly, it showed that the vaccination rates *increased* with increasing percentages of Native Americans (Indians) and Hispanics ($\beta_{9}$ = 0.202, *p* < 0.001; $\beta_{9}$ = 0.057, *p* = 0.008, respectively) and *decreasing* rates of the Black population ($\beta_{9}$ = −0.419, *p* < 0.001), whereas the White and Asian population effects were not significant in predicting a county’s vaccination rate. In terms of the role of socioeconomic factors, this suggests principally that Native Americans and Hispanics would be more likely to seek and accept the COVID-19 vaccination than their Black counterparts, while such likelihood was not evident among Whites and other minorities. In Figure 4b, the 95% confidence interval was mapped to illustrate such race/ethnicity–vaccination relationship, displaying statistically significant and positive effects on the change in the vaccination rate from Indians and the Hispanic group, contrasting with the significant but opposite effect contributed by the Black citizens. Equally, Tables S1 and S2 summarizing the analytical results over different age variables revealed the consistent connection between vaccination rates and the race/ethnicity variables.

Finally, a couple of additional points are worth noting. In Table 3, model M3.2, including Black as a race variable, explained 48.8% of the variation in the vaccination rate. The results imply racial disparities, particularly among Blacks, regarding COVID-19 vaccination coverage, as claimed by Kricorian and Turner [35]. For all the models adjusted by age and race variables, we observed consistent results for the county’s state, Democratic affiliation, and income per capita as significant factors affecting vaccination rates. However, the rural residence of county citizens was found to be negatively correlated with the vaccination rate in the Native American model (M3.4) in Table 3, showing a *decreasing* rate of vaccination among those who dwell in the county with more and more remote rural areas ($\beta_{5}$ = −0.023, *p* = 0.046). 

## 4. Discussion and Policy Implications

Since the outbreak of COVID-19, as all people around the globe are under siege, policymakers from almost all levels and fields have tried to work and race against time to cope with and defeat such an unprecedented ‘deadly enemy.’ After two ‘long’ years of battle, many countries still see COVID-19 as a national threat needing continuous efforts to fight it, while believing that a change in life seems inevitable in the long run. After the individual (national) and collective (international) research and development effort to develop the COVID-19 vaccines, the launch of vaccination was like the dawn of a new day giving hope to the global community, although the global vaccine coverage is far from complete. In the US, the cross-state ‘vaccination race’ started in early 2021. While the entire nation continues to work industriously to increase the national vaccination coverage, factors affecting COVID-19 vaccination across the states are proved to be multifaceted. Hence, the policies proposed for vaccination promotion should be pragmatic and strategic, targeting both factor-specific schemes and taking a generalized approach.

*“No one is safe from COVID-19 until everyone is safe.”* [3]. Indeed, getting COVID-19 under control is revealed to be when a population is by and large vaccinated, from every individual in a country to the entire population worldwide. Herd immunity and ‘vaccine equity’ across countries are crucial, while practical actions to increase vaccination rates are the real need. Within the US, ‘vaccine equity’ may only be a reality when citizens of all races and ethnicities are reached for vaccination. Policymakers are recommended to understand fundamental ethnic culture and differences while practicing no racial discrimination to promote civilian trust and expand vaccination.

*Political preference matters! (Or, should it not matter?)* As indicated in the empirical findings, county-based politics faces a ‘political divide,’ showing Democratic voters tending to receive COVID-19 vaccination more than their opponents. Is it not time to think beyond politics? The Republican political leaders, along with their Democratic and other political rivals, need to persuade their respective followers that living or being alive is and should be above all things. Policymakers are therefore advised to work with politicians of all parties to communicate with their supporters about life-sustaining protection through vaccination as the Number One priority. 

*Personal income leads to vaccination variation.* In this study, per-capita income appears to affect COVID-19 vaccination across counties when citizens facing economic and financial struggles tend to delay or decline immunization. Even if the cost of COVID-19 vaccination across the US is generally covered by citizens’ medical insurance or sponsored by the state or federal governments, low-income or underprivileged individuals and households should not feel stressed when getting vaccinated. Other socioeconomic factors, such as occupation relevant to personal income, also need further discussion. Studies have found that COVID-19 has exacerbated income and socioeconomic inequalities, which implies that a proper vaccination policy must be designed to support various occupations such as environmental services workers who appear to be COVID-19-vulnerable [37]. Policymakers are suggested to provide equal access, including assistance with vaccination registration and transportation logistics, especially when such resources are unavailable or less accessible to those citizens.

## 5. Conclusions

In conclusion, it is found that U.S. county-level vaccination rates were significantly associated with the percentage of Democrat votes, per capita income, and the state to which the county belongs. Pertaining to age differences, an increased county vaccination rate was observed as the percentage of the elderly population increased, whereas it decreased as the share of the county working class population aged 18 to 64 increased. County-level differences in racial and ethnic COVID-19 vaccination acceptance prevailed, showing that vaccine acceptance decreased with an increasing rate of the Black population, counter to an increasing vaccination rate in counties with more Native Americans and Hispanics. This study serves to alert policymakers to the potential vaccination impediments while it advises the public that for the goal of pandemic alleviation and crisis recovery increasing vaccination would presumably be fundamental.

## Figures and Tables

**Table 1:** Variables and data sources used in the study of COVID-19 vaccination rates.

Factor	Variable	Description	Data Source
State	*State*	States (Arizona, Colorado, New Mexico, Oklahoma, and Texas)	
Employment status	*Unemployment*	Unemployment rate in 2019	Bureau of Labor Statistics published by Economic Research Service of USDA ^a^ https://data.ers.usda.gov/reports.aspx?ID=17828 (accessed on 8 June 2021)
Political choice	*Democrat*	Percent of democrat votes, 2020 election result	Politico https://www.politico.com/2020-election/results/ (accessed on 15 June 2021)
	*Democrat_1*	Democrat = 1, Republican = 0	
Age	*Age_1417*	Percent of county resident population aged between 14 and 17	2010 US Census (1 April 2010 to 1 July 2019) https://www.census.gov/data/tables/time-series/demo/popest/2010s-counties-detail.html (accessed on 15 June 2021)
	*Age_1864*	Percent of county resident population aged between 18 and 64	
	*Age_65over*	Percent of county resident population aged 65 years and over	
	*Age_14over*	Percent of county resident population aged 14 years and over	
	*Age_18over*	Percent of county resident population aged 18 years and over	
	*Age_median*	Median age of county resident population	
Occupation	*FarmWorker*	Percent of workers hired farm labor	Bureau of Economic Analysis 2019 https://apps.bea.gov/iTable/iTable.cfm?reqid=70&step=1&acrdn=6 (accessed on 10 August 2021)
Area of residence	*Rural_pct*	Percent of the county population living in rural areas	2010 US Census https://apps.bea.gov/iTable/iTable.cfm?reqid=70&step=1&acrdn=6 (accessed on 15 June 2021)
	*Urban_1*	Urban = 1 if Rural_pct ≤ 50, Rural = 0 if Rural_pct > 50	
Education	*HS graduate*	Percent of county population who are a high school graduates or higher (5-year estimate) for the population 18 years old and over	Federal Reserve Economic Data 2019 https://fred.stlouisfed.org/release/tables?rid=330&eid=391443 (accessed on 12 July 2021)
Income	*Income*	Per capita personal income	Bureau of Economic Analysis 2019 https://apps.bea.gov/iTable/iTable.cfm?reqid=70&step=1&acrdn=6 (accessed on 15 June 2021)
Race/ethnicity	*White*	Percent of county population by race (White)	2010 US Census https://www.census.gov/data/tables/time-series/demo/popest/2010s-counties-detail.html (accessed on 15 June 2021)
	*Black*	Percent of county population by race (Black)	
	*Asian*	Percent of county population by race (Asian)	
	*Indian*	Percent of county population by race (Indian)	
	*Other*	Percent of county population by race (other)	
	*Hispanic*	Percent of county population by Hispanic origin (Hispanic)	
	*Non-Hispanic*	Percent of county population by non-Hispanic origin (non-Hispanic)	
Vaccination	*Vaccination rate*	Percent of people who are fully vaccinated based on the jurisdiction and county where recipient lives as of 1 May 2021	CDC ^b^ https://data.cdc.gov/Vaccinations/COVID-19-Vaccinations-in-the-United-States-County/8xkx-amqh (accessed on 1 June 2021), Texas vaccination data https://data.democratandchronicle.com/covid-19-vaccine-tracker/texas/48/ (accessed on 22 June 2021)

^a^ USDA: United States Department of Agriculture, ^b^ CDC: Centers for Disease Control and Prevention.

**Table 2:** Preliminary summary (mean ± sd/frequency (%)) of counties (*N* = 443) and the information by state.

	All *	AZ	CO	NM	OK	TX
*N*	443	15	64	33	77	254
Unemployment (%)	3.61 ± 1.47	6.64 ± 3.28	2.74 ± 0.81	5.44 ± 1.64	3.25 ± 0.92	3.51 ± 1.09
Democrat (%)	29.07 ± 17.38	62.50 ± 7.24	41.73 ± 18.59	44.79 ± 16.91	20.27 ± 7.93	24.53 ± 14.15
Democrat_1	75 (16.9)	15 (100)	24 (37.5)	14 (42.4)	0 (0)	22 (8.7)
Age (%)						
14–17 years old	5.29 ± 0.89	5.02 ± 0.88	4.67 ± 0.85	4.93 ± 0.97	5.39 ± 0.48	5.48 ± 0.90
18–64 years old	57.88 ± 4.22	55.95 ± 5.31	60.01 ± 5.33	56.40 ± 4.28	57.69 ± 2.87	57.70 ± 4.00
≥ 65 years old	19.15 ± 5.56	21.70 ± 7.98	20.02 ± 5.59	22.15 ± 7.67	18.63 ± 2.85	18.55 ± 5.53
≥ 14 years old	82.32 ± 3.16	82.67 ± 3.19	84.71 ± 3.30	83.49 ± 3.43	81.71 ± 1.52	81.74 ± 3.15
≥ 18 years old	77.03 ± 3.88	77.65 ± 4.03	80.04 ± 4.03	78.55 ± 4.29	76.32 ± 1.82	76.25 ± 3.82
Median (years old)	40.31 ± 6.02	41.23 ± 8.35	42.94 ± 6.14	42.65 ± 8.06	39.56 ± 3.38	39.52 ± 5.92
Farm Worker (%)	5.93 ± 5.96	1.58 ± 1.83	5.52 ± 6.98	4.67 ± 5.91	5.82 ± 4.65	6.48 ± 6.10
Rural_pct (%)	56.13 ± 31.67	34.11 ± 19.75	58.68 ± 36.63	48.42 ± 30.54	63.64 ± 26.13	55.52 ± 31.90
Urban_1	202 (45.6)	12 (80)	30 (46.9)	21 (63.6)	21 (27.3)	118 (46.5)
HS graduate (%)	83.64 ± 7.97	84.48 ± 5.32	91.16 ± 4.84	84.36 ± 5.42	86.11 ± 3.74	80.85 ± 8.46
Income ($)	45,928 ± 13,399	39,197 ± 5887	53,080 ± 19,436	41,800 ± 9196	41,248 ± 8238	46,479 ± 12,720
Race (%)						
White	86.46 ± 10.38	78.32 ± 19.40	91.83 ± 4.13	84.94 ± 15.84	77.17 ± 10.45	88.61 ± 7.49
Black	5.12 ± 5.62	2.36 ± 1.85	2.01 ± 2.41	1.96 ± 1.32	3.80 ± 3.49	6.88 ± 6.46
Asian	1.31 ± 1.76	1.57 ± 1.15	1.48 ± 1.42	1.24 ± 1.12	0.98 ± 0.98	1.35 ± 2.08
Indian	4.31 ± 8.17	15.10 ± 20.69	2.22 ± 2.06	9.32 ± 16.14	11.79 ± 8.40	1.28 ± 0.51
Other	2.80 ± 1.95	2.66 ± 0.72	2.47 ± 0.73	2.55 ± 0.63	6.26 ± 2.17	1.88 ± 0.68
Ethnicity (%)						
Hispanic	29.63 ± 22.40	31.71 ± 20.83	20.16 ± 13.54	48.69 ± 17.05	9.71 ± 7.58	35.46 ± 22.97
Non-Hispanic	70.37 ± 22.40	68.29 ± 20.83	79.84 ± 13.54	51.31 ± 17.05	90.29 ± 7.58	64.54 ± 22.97
Vaccination rate (%) (as of 1 May 2021)	24.59 ± 6.87	32.81 ± 7.96	19.63 ± 7.14	26.49 ± 9.64	24.68 ± 4.46	25.08 ± 6.12

* All counties in five states; Arizona (AZ), Colorado (CO), New Mexico (NM), Oklahoma (OK), and Texas (TX).

**Table 3:** Associations between vaccination rates and the counties’ characteristics in the regression analyses.

	(M1)	(M2)	(M3.1)	(M3.2)	(M3.3)	(M3.4)	(M3.5)
	Model 1	Model 1+ Age_65over	Model 1+ Age_65over + Race (White)	Model 1+ Age_65over + Race (Black)	Model 1+ Age_65over + Race (Asian)	Model 1+ Age_65over + Race (Indian)	Model 1+ Age_65over + Race (Hispanic)
R-squared	0.382	0.404	0.405	0.488	0.405	0.439	0.414
State (NM *)							
AZ	2.210 (0.206)	2.116 (0.218)	2.172 (0.207)	1.542 (0.334)	2.181 (0.205)	1.310 (0.435)	3.440 (0.053)
CO	**−6.287** (<0.001)	**−5.778** (<0.001)	**−5.901** (<0.001)	**−5.582** (<0.001)	**−5.736** (<0.001)	**−4.815** (<0.001)	**−4.861** (<0.001)
OK	**4.657** (<0.001)	**5.582** (<0.001)	**5.897** (<0.001)	**6.750** (<0.001)	**5.501** (<0.001)	**4.692** (<0.001)	**7.124** (<0.001)
TX	**3.453** (0.004)	**4.024** (0.001)	**4.076** (0.001)	**6.985** (<0.001)	**3.896** (0.001)	**5.178** (<0.001)	**4.776** (<0.001)
Unemployment	0.285 (0.251)	0.105 (0.672)	0.151 (0.553)	0.327 (0.158)	0.112 (0.651)	−0.121 (0.622)	0.127 (0.608)
Democrat	**0.242** (<0.001)	**0.243** (<0.001)	**0.247** (<0.001)	**0.260** (<0.001)	**0.237** (<0.001)	**0.232** (<0.001)	**0.222** (<0.001)
Farm Worker	0.077 (0.220)	0.019 (0.764)	0.019 (0.765)	−0.002 (0.968)	0.014 (0.827)	0.012 (0.847)	−0.017 (0.788)
Rural_pct	0.004 (0.711)	−0.013 (0.278)	−0.012 (0.319)	−0.012 (0.274)	−0.010 (0.409)	**−0.023** (0.046)	−0.005 (0.692)
HS graduate	−0.012 (0.765)	−0.063 (0.136)	−0.053 (0.222)	0.026 (0.525)	−0.072 (0.097)	−0.073 (0.076)	0.033 (0.550)
Income	**8.077e-05** (<0.001)	**8.982e-05** (<0.001)	**8.658e-05** (<0.001)	**6.124e-05** (0.004)	**8.77e-05** (<0.001)	**1.009e-04** (<0.001)	**9.199e-05** (<0.001)
Age_65over		**0.245** (<0.001)	**0.230** (<0.001)	**0.149** (0.011)	**0.253** (<0.001)	**0.302** (<0.001)	**0.262** (<0.001)
Race/ethnicity			0.027 (0.395)	**−0.419** (<0.001)	0.161 (0.369)	**0.202** (<0.001)	**0.057** (0.008)

* refers to a reference category. All significant estimates are expressed in bold and *p*-values in parentheses.

## Data Availability

The dataset supporting the conclusions of this article is available in the Mendeley Data repository, https://data.mendeley.com/datasets/vwfzpgbs2h/2, accessed on 15 June 2021.

## Supplementary Materials

The following supporting information can be downloaded at: https://www.mdpi.com/article/10.3390/epidemiologia3040038/s1, Table S1: Associations between vaccination rate and the counties’ characteristics in the regression analyses, including the age group of 18–64 years; Table S2: Associations between vaccination rate and the counties’ characteristics in the regression analyses, including the median age group.
