# Supplementary material for: COVID-19 Vaccination: Sociopolitical and Economic Impact in the United States
Source: Epidemiologia (Basel). 2022 Nov 8;3(4):502–17. doi: 10.3390/epidemiologia3040038 (PMC9680412; doi:10.3390/epidemiologia3040038)
Supplement: Supplementary file 1 [file epidemiologia-03-00038-s001.zip › epidemiologia-1962972-supplementary.pdf]

Table S1. Associations between vaccination rate and the counties' characteristics in the regression analyses, including the age group of 18-64 years.

	(M1)<br>Model 1	(M2)<br>Model 1<br>+ Age_1864	(M3.1)<br>Model 1<br>+ Age_1864<br>+ Race (White)	(M3.2)<br>Model 1<br>+ Age_1864<br>+ Race (Black)	(M3.3)<br>Model 1<br>+ Age_1864<br>+ Race (Asian)	(M3.4)<br>Model 1<br>+ Age_1864<br>+ Race (Indian)	(M3.5)<br>Model 1<br>+ Age_1864<br>+ Race (Hispanic)
R-squared	0.382	0.399	0.401	0.482	0.400	0.427	0.414
State (NM*)							
AZ	2.210	1.828	1.922	1.454	1.877	1.096	2.818
	(0.206)	(0.290)	(0.267)	(0.366)	(0.278)	(0.518)	(0.117)
CO	**-6.287**	**-5.677**	**-5.841**	**-5.670**	**-5.636**	**-4.869**	**-5.063**
	(<0.001)	(<0.001)	(<0.001)	(<0.001)	(<0.001)	(<0.001)	(<0.001)
OK	**4.657**	**5.026**	**5.468**	**6.343**	**4.939**	**4.085**	**6.087**
	(<0.001)	(<0.001)	(<0.001)	(<0.001)	(<0.001)	(0.002)	(<0.001)
TX	**3.453**	**3.813**	**3.895**	**6.816**	**3.694**	**4.754**	**4.315**
	(0.004)	(0.001)	(0.001)	(<0.001)	(0.002)	(<0.001)	(<0.001)
Unemployment	0.285	0.086	0.146	0.369	0.092	-0.096	0.121
	(0.251)	(0.731)	(0.570)	(0.119)	(0.714)	(0.700)	(0.631)
Democrat	**0.242**	**0.254**	**0.257**	**0.263**	**0.249**	**0.244**	**0.237**
	(<0.001)	(<0.001)	(<0.001)	(<0.001)	(<0.001)	(<0.001)	(<0.001)
Farm Worker	0.077	0.015	0.015	0.010	0.010	0.014	-0.006
	(0.220)	(0.819)	(0.815)	(0.866)	(0.877)	(0.821)	(0.929)
Rural_pct	0.004	-0.003	-0.002	-0.004	0.00004	-0.009	0.004
	(0.711)	(0.813)	(0.845)	(0.687)	(0.997)	(0.408)	(0.711)
HS graduate	-0.012	-0.014	-0.006	0.057	-0.021	-0.012	0.059
	(0.765)	(0.729)	(0.891)	(0.138)	(0.615)	(0.752)	(0.287)
Income	**8.077e-05**	**7.919e-05**	**7.592e-05**	**5.479e-05**	**7.7e-05**	**8.698e-05**	**8.041e-05**
	(<0.001)	(<0.001)	(0.001)	(0.010)	(0.001)	(<0.001)	(<0.001)
**Age_1864**		**-0.265**	**-0.244**	-0.064	**-0.273**	**-0.291**	**-0.252**
		(0.001)	(0.002)	(0.204)	(<0.001)	(<0.001)	(0.001)
Race/ethnicity			0.034	**-0.426**	0.143	**0.179**	0.041
			(0.278)	(<0.001)	(0.428)	(<0.001)	(0.054)

* refers to a reference category. All significant estimates are expressed in bold and p-values in parentheses.

Table S2. Associations between vaccination rate and the counties' characteristics in the regression analyses, including the median-age group.

	(M1)<br>Model 1	(M2)<br>Model 1<br>+ Age_median	(M3.1)<br>Model 1<br>+ Age_median<br>+ Race (White)	(M3.2)<br>Model 1<br>+ Age_median<br>+ Race (Black)	(M3.3)<br>Model 1<br>+ Age_median<br>+ Race (Asian)	(M3.4)<br>Model 1<br>+ Age_median<br>+ Race (Indian)	(M3.5)<br>Model 1<br>+ Age_median<br>+ Race (Hispanic)
R-squared	0.382	0.402	0.403	0.489	0.402	0.435	0.413
State (NM*)							
AZ	2.210	2.332	2.388	1.675	2.378	1.594	3.798
	(0.206)	(0.176)	(0.166)	(0.294)	(0.168)	(0.343)	(0.033)
CO	**-6.287**	**-6.045**	**-6.178**	**-5.725**	**-6.026**	**-5.171**	**-5.052**
	(<0.001)	(<0.001)	(<0.001)	(<0.001)	(<0.001)	(<0.001)	(<0.001)
OK	**4.657**	**5.490**	**5.908**	**6.764**	**5.427**	**4.593**	**7.206**
	(<0.001)	(<0.001)	(<0.001)	(<0.001)	(<0.001)	(<0.001)	(<0.001)
TX	**3.453**	**3.842**	**3.927**	**6.932**	**3.751**	**4.920**	**4.668**
	(0.004)	(0.001)	(0.001)	(<0.001)	(0.002)	(<0.001)	(<0.001)
Unemployment	0.285	0.162	0.214	0.355	0.169	-0.044	0.183
	(0.251)	(0.513)	(0.394)	(0.122)	(0.495)	(0.857)	(0.454)
Democrat	**0.242**	**0.240**	**0.245**	**0.258**	**0.236**	**0.228**	**0.216**
	(<0.001)	(<0.001)	(<0.001)	(<0.001)	(<0.001)	(<0.001)	(<0.001)
Farm Worker	0.077	0.052	0.049	0.016	0.050	0.053	0.013
	(0.220)	(0.405)	(0.432)	(0.789)	(0.428)	(0.385)	(0.832)
Rural_pct	0.004	-0.017	-0.016	-0.016	-0.015	**-0.028**	-0.009
	(0.711)	(0.174)	(0.212)	(0.166)	(0.245)	(0.024)	(0.456)
HS graduate	-0.012	-0.075	-0.062	0.015	-0.080	-0.086	0.026
	(0.765)	(0.087)	(0.167)	(0.712)	(0.072)	(0.043)	(0.636)
Income	**8.077e-05**	**8.225e-05**	**7.874e-05**	**5.649e-05**	**8.069e-05**	**9.130e-05**	**8.414e-05**
	(<0.001)	(<0.001)	(0.001)	(0.007)	(<0.001)	(<0.001)	(<0.001)
**Age_median**		**0.222**	**0.207**	**0.149**	**0.224**	**0.269**	**0.248**
		(<0.001)	(0.001)	(0.007)	(<0.001)	(<0.001)	(<0.001)
Race/ethnicity			0.034	**-0.424**	0.105	**0.196**	**0.062**
			(0.272)	(<0.001)	(0.555)	(<0.001)	(0.004)

* refers to a reference category. All significant estimates are expressed in bold and p-values in parentheses.
